# Establishment of a stable transfection system for genetic manipulation of *Babesia gibsoni*

**DOI:** 10.1186/s13071-018-2853-1

**Published:** 2018-04-23

**Authors:** Mingming Liu, Paul Franck Adjou Moumouni, Masahito Asada, Hassan Hakimi, Tatsunori Masatani, Patrick Vudriko, Seung-Hun Lee, Shin-ichiro Kawazu, Junya Yamagishi, Xuenan Xuan

**Affiliations:** 1 National Research Center for Protozoan Diseases, Obihiro University of Agriculture and Veterinary Medicine, Inada-cho, Obihiro, Hokkaido 080-8555 Japan; 2 Department of Protozoology, Institute of Tropical Medicine, Nagasaki University, Sakamoto 1-12-4, Nagasaki, 852-8523 Japan; 3 Transboundary Animal Diseases Research Center, Joint Faculty of Veterinary Medicine, Kagoshima University, 1-21-24 Korimoto, Kagoshima, 890-0065 Japan; 4 Research Center for Zoonosis Control, Hokkaido University, North 20, West 10 Kita-ku, Sapporo, Hokkaido 001-0020 Japan; 5 Global Station for Zoonosis Control, GI-CoRE, Hokkaido University, North 20, West 10 Kita-ku, Sapporo, Hokkaido 001-0020 Japan

**Keywords:** Apicomplexa, *Babesia gibsoni*, Stable transfection, Homologous recombination

## Abstract

**Background:**

Genetic manipulation techniques, such as transfection, have been previously reported in many protozoan parasites. In *Babesia*, stable transfection systems have only been established for bovine *Babesia* parasites. We recently reported a transient transfection system and the selection of promoter candidates for *Babesia gibsoni*. The establishment of a stable transfection system for *B. gibsoni* is considered to be urgent to improve our understanding of the basic biology of canine *Babesia* parasites for a better control of babesiosis.

**Results:**

GFP-expressing parasites were observed by fluorescence microscopy as early as two weeks after drug selection, and consistently expressed GFP for more than 3 months without drug pressure. Genome integration was confirmed by PCR, sequencing and Southern blot analysis.

**Conclusions:**

We present the first successful establishment of a stable transfection system for *B. gibsoni*. This finding will facilitate functional analysis of *Babesia* genomes using genetic manipulation and will serve as a foundation for the development of tick-*Babesia* and host-*Babesia* infection models.

**Electronic supplementary material:**

The online version of this article (10.1186/s13071-018-2853-1) contains supplementary material, which is available to authorized users.

## Background

*Babesia gibsoni* is a tick-borne intraerythrocytic apicomplexan parasite which causes canine babesiosis [1]. During the asexual phase of its life-cycle occurring in the vertebrate host, *B. gibsoni* causes progressive anemia, remittent fever, hemoglobinuria, marked splenomegaly, hepatomegaly and sometimes death [2]. *Babesia gibsoni* has a global distribution, with considerable a significant impact on canine health [3].

The difficulties in identifying *B. gibsoni* virulence factors and developing successful therapies have been attributed in part to the lack of genetic manipulation tools [4]. Transfection systems have been established for several apicomplexan parasites, such as *Cryptosporidium parvum* [5], *Plasmodium falciparum* [6], *Toxoplasma gondii* [7], *Theileria annulata* [8] and *T. parva* [9]. Among *Babesia* species, transient and stable transfection systems have been reported for *B. bovis* [10], *B. ovata* [11] and *B. bigemina* [12]. For *B. gibsoni*, only two reports have described transient transfection systems [13, 14]. *Babesia gibsoni elongation factor-1 alpha* (*Bg 5′-ef-1α*) promoter, Program FA113 of AMAXA 4D Nucleofector™ and Lonza buffer SF successfully supported the expression of reporter genes [13]. In addition, among the 12 promoter candidates tested, *Bg 5′-actin* was found to be the most active promoter [14]. Similar to *B. bovis* [15], the development of a stable transfection system for *B. gibsoni* parasites requires a drug selection system and an integration target. The WR99210/human dihydrofolate reductase gene (*hdhfr*) selection system and double cross-over homologous recombination locus have previously been successfully used for *B. bovis* [16] and *B. ovata* [11] stable transfection.

In this study, in order to establish *B. gibsoni* stable transfection, we investigated whether stable transfection of GFP-expressing *B. gibsoni* could be achieved using *hdhfr* as a selectable marker under the control of the *Bg 5′-ef-1α* (IG-B) and *Bg 5′-actin* promoters, and *ef-1α* locus as the integration target.

## Methods

### Parasite culture

In this study, *B. gibsoni* Oita strain [17] was cultured *in vitro* in 24-well culture plates (Thermo Fisher Scientific, Waltham, MA, USA) at 37 °C in humidified CO_2_ (5%) and O_2_ (5%) incubator (BIO-LABO, Tokyo, Japan). The parasite was cultured in 10% canine erythrocytes suspended in RPMI-1640 supplemented with 20% canine serum.

### Evaluation of *B. gibsoni* sensitivity to WR99210

*Babesia gibsoni* was cultured *in vitro* in 96-well culture plates with 100 μl RPMI-1640 medium containing 10% canine erythrocytes supplemented with 20% dog serum and different concentrations of WR99210 (0.1, 0.5, 1, 5, 10 and 100 nM). For each drug concentration, parasites were cultured in triplicate wells and the culture medium was replaced daily. Parasitemia was calculated on day 3 by examining 3000 RBCs of a prepared thin blood smear stained with Giemsa solution.

### Plasmid constructs

**Fig. 1:**
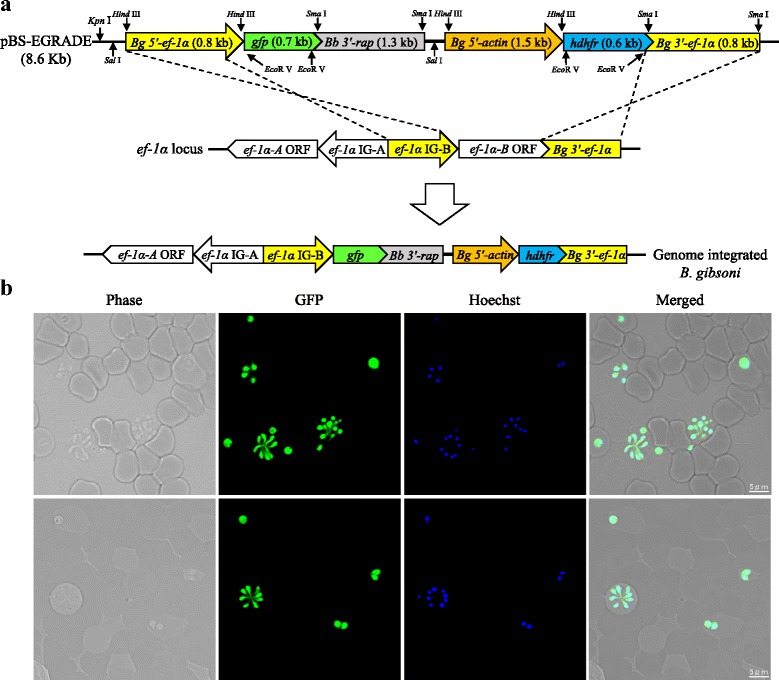
Schematic diagram of GFP-expressing plasmid (pBS-EGRADE) construct and fluorescence microscopy images of stably expressing GFP *B. gibsoni*. **a** Plasmid construct of pBS-EGRADE showing the recombination sites for integration into *ef-1α* locus by double cross-over homologous recombination. The restriction site for linearization (*Kpn* I) is shown. **b** Fluorescence microscopy images of stable GFP-expressing *B. gibsoni*. Merged panel shows overlap of GFP and Hoechst (parasite nuclei) fluorescence. The parasite nucleus was stained with Hoechst 33342

The schematic diagram of the plasmid used in this study (pBS-EGRADE) is shown in Fig. 1a. The reporter gene and drug selection gene cassettes were separated in order to drive *gfp* and *hdhfr* with *Bg 5′-ef-1α* (IG-B) and *Bg 5′-actin* promoters, respectively. *Bg 5′-ef-1α* (IG-B) and *Bg 3′-ef-1α* were used as recombination sites cloned into the upstream and downstream of the *gfp* and *hdhfr* genes, respectively. All the PCR primer pairs used for plasmid construction are listed in Table 1 and restriction sites are underlined. The constructed plasmid was purified using Qiagen® Plasmid Maxi Kit (Qiagen, Hilden, Germany) according to the manufacturer's instructions, and was confirmed by sequencing before transfection. The sequence of pBS-EGRADE plasmid was deposited in the GenBank database under the accession number MG913246.

**Table 1:** List of primers used in this study

Primer	Sequence (5'–3')^a^
*Bg 5′-ef-1α*-F (*Hind* III)	GACGGTATCGATAAGCTTCACTGTATAACGGATGAAGGT
*Bg 5′-ef-1α*-R (*Hind* III)	CACCATGATATCAAGCTTTTTGGTAAAGGTTGACGATA
GFP-F (*Eco*R V)	ATCGATAAGCTTGATATCATGGTGAGCAAGGGCGA
GFP-R (*Eco*R V)	CTGCAGGAATTCGATATCTTACTTGTACAGCTCGTCCATG
*Bb 3′-rap*-F (*Sma* I)	GAATTCCTGCAGCCCGGGGATGAGATGCGTTTATAATGGC
*Bb 3′-rap*-R (*Sma* I)	ACTAGTGGATCCCCCGGGCCTACGAACGATATGTCAAAGAG
*Bg 5′-actin*-F (*Hind* III)	GACGGTATCGATAAGCTTCCAGTAAAAAGTGACTACCATA
*Bg 5′-actin*-R (*Hind* III)	AACCATGATATCAAGCTTTTTGATAACGTAATAGCTCTGTA
hDHFR-F (*Eco*R V)	ATCGATAAGCTTGATATCATGGTTGGTTCGCTAAAC
hDHFR-R (*Eco*R V)	CTGCAGGAATTCGATATCTTAATCATTCTTCTCATATACTTC
*Bg* 3′-*ef-1α*-F (*Sma* I)	GAATTCCTGCAGCCCGGGAGCTGATTATTTCGTGTTAACT
*Bg* 3′-*ef-1α*-R (*Sma* I)	ACTAGTGGATCCCCCGGGGATTGGTAGTATTTGTCGTCAT
EGR-F (*Sal* I)	CCCCCCCTCGAGGTCGACCACTGTATAACGGATGAAGGT
EGR-R (*Sal* I)	CTTATCGATACCGTCGACCCTACGAACGATATGTCAAAGAG
Integ-F	TAGCAGCCAAGCGAGATA
Integ-R	CAACTTAGATTGATCGGTG
Probe-GFP-F	ATGGTGAGCAAGGGCGA
Probe-GFP-R	TTACTTGTACAGCTCGTCCATG
Probe-3′-*ef-1α*-F	ATCCCCTGTCTCAATGG
Probe-3′-*ef-1α*-R	GATTGGTAGTATTTGTCGTCA

^a^Restriction enzyme sites are underlined

### Transfection of parasites

*Babesia gibsoni*-infected red blood cells (iRBCs) were pre-treated as previously described [13]. Transfection was conducted using 20 μg of linearized pBS-EGRADE plasmid. The plasmid-iRBCs mixtures were transfected using Lonza buffer SF and program FA113 of Amaxa 4D Nucleofector™ device (Lonza, Cologne, Germany) and immediately transferred into a preheated culture containing 10% fresh RBCs. To avoid the rapid *in vitro* aging of canine erythrocytes, transfected parasites were subcultured every week and supplemented with fresh RBCs. To select GFP-expressing transgenic parasites, 10 nM WR99210 was added to the culture medium two days after transfection. After 4 weeks of drug selection, the parasite population was cloned in a 96-well culture plate using limiting dilution as previously described [16].

### PCR characterization of GFP-expressing *B. gibsoni*

Three sets of primers (Table 1) were used to confirm the integration of pBS-EGRADE into *B. gibsoni ef-1α* locus. Primer pair Integ-F and GFP-R was used to amplify a 1.6 kb DNA fragment to confirm the 5′ recombination. Primer pair hDHFR-F and Integ-R was used to amplify a 2.0 kb DNA fragment to examine the 3′ recombination whereas primer pair GFP-F and hDHFR-R was used to amplify a 4.1 kb DNA fragment to detect the insertion region. The DNA fragments amplified were confirmed by sequencing.

### Southern blot analysis

Two micrograms of genomic DNA from wild type (WT) and genome integrated (GI) *B. gibsoni* were digested overnight with 20 units of *Sca* I and *Sph* I. The digestion products were separated by agarose gel electrophoresis, transferred onto Hybond N^+^ membrane (GE Healthcare, Buckinghamshire, UK) then hybridized with labeled probes using an AlkPhos Direct Kit (GE Healthcare, Buckinghamshire, UK) according to the manufacturer′s instructions. Two probes corresponding to the complete open reading frame (ORF) of *gfp* and the 0.4 kb length of *Bg 3′-ef-1α* fragment, respectively, were used. The primer pairs used to amplify the probes are listed in Table 1. Probe signal was detected using a CDP-star detection reagent (GE Healthcare).

### Growth curves

WT and GI parasites were continuously cultured from approximately 0.5% parasitemia by sub-culturing every 3 days for two generations. Parasitemia were monitored daily by examining 3000 RBCs with Giemsa staining.

## Results

### *Babesia gibsoni* sensitivity to WR99210

WR99210 successfully inhibited the growth of *B. gibsoni in vitro* at a nanomolar concentration (Additional file 1: Figure S1). The calculated IC_50_ was 1.1 nM, and 10 nM WR99210 completely inhibited the growth of *B. gibsoni*. Thus, 10 nM WR99210 was used for the selection of transfected parasites.

### Establishment of stable GFP expression in *B. gibsoni*

**Fig. 2:**
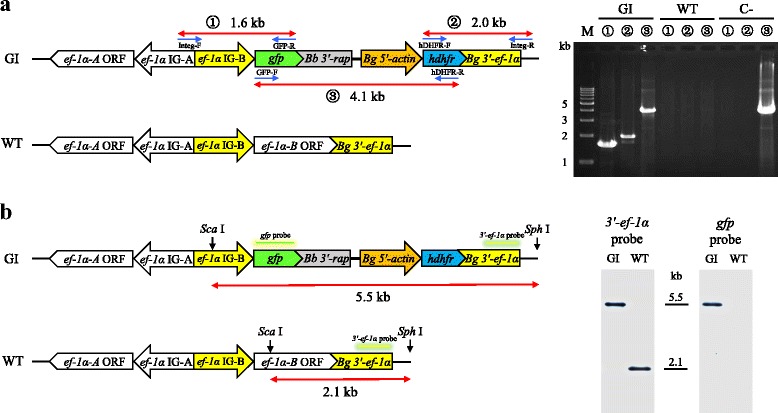
Confirmation of integration of pBS-EGRADE into the *ef-1α* locus. **a** Schematic diagram and results of PCR to confirm the integration of pBS-EGRADE into the *ef-1α* locus. PCR-1, -2 and -3 were done with primer sets Integ-F/GFP-R, hDHFR-F/Integ-R and GFP-F/hDHFR-R, respectively. **b** Schematic diagram and Southern blot analysis to confirm the integration of pBS-EGRADE into *ef-1α* locus. Two μg of samples genomic DNA were digested with *Sca* I and *Sph* I, and hybridized with *3′-ef-1α* and *gfp* probes. *Abbreviations*: GI, genome-integrated; WT, wild type; C-, pBS-EGRADE plasmid control

GFP-expressing parasites emerged as early as two weeks after drug selection with 10 nM WR99210. The parasite population was cloned by limiting dilution and consistently expressed GFP for more than 3 months without drug pressure (Fig. 1b). After obtaining parasite clonal lines, the correct integration of pBS-EGRADE into the *ef-1α* locus was confirmed by the results of both PCR and Southern blot analysis. The PCR-1, -2 and -3 primer pairs successfully amplified 1.6, 2.0 and 4.1 kb DNA fragments, respectively, using DNA template from one clonal line named GI parasite (Fig. 2a) and the amplified DNA fragments were validated by sequencing. The sequences of the above DNA fragments were deposited in the GenBank database under the accession numbers MH087225-MH087227. No amplicons were obtained with DNA template from the WT parasite. In Southern blot analysis, both *gfp* and *3′-ef-1α* probes detected a single 5.5 kb band for GI parasite, while the *3′-ef-1α* probe detected a single 2.1 kb band, and the *gfp* probe did not detect any band for the WT parasite (Fig. 2b). In addition, the growth curves of WT and GI parasites showed high similarity (Additional file 2: Figure S2).

## Discussion

Transfection systems improve our understanding of the molecular biology of parasites and pave the way for genetic manipulation [18]. The application of transfection systems can also lead to a better understanding of the mechanisms underlying drug resistance, host-parasite interactions, and provide novel information for vaccine development and drug target discovery [19]. Currently, there is a lack of techniques for the genetic manipulation of *B. gibsoni*. In order to fill this gap, we describe herein the development of a stable transfection system for *B. gibsoni*.

In this study, we employed a WR99210/*hdhfr* selection system for *B. gibsoni* stable transfection. The IC_50_ of WR99210 against *B. gibsoni* was 1.1 nM (Additional file 1: Figure S1), which is similar to *B. bovis* (1 nM) [16] and almost twice that of the one reported for *B. ovata* (0.56 nM) [11]. The transfected parasite selected with WR99210/*hdhfr* emerged as early as two weeks after adding the drug, indicating the suitability of this selection system for stable transfection of *B. gibsoni*. *Babesia bovis 3′-rap* was successfully used as terminator in this study (Fig. 1a). This result is consistent with our previous work [14], confirming that *Bb 3′-rap* heterologous terminator is fully functional in *B. gibsoni*. These findings provide considerable flexibility in the construction of plasmid vectors to be used for transfection systems in *Babesia* species. The cloned GI parasite stably expressed GFP (Fig. 1b) and PCR amplicons (Fig. 2a) and Southern blot analyses (Fig. 2b) indicated that pBS-EGRADE was integrated into *B. gibsoni* genome by homologous recombination as expected. In addition, the growth of GI parasite was comparable with that of the WT parasite (Additional file 2: Figure S2). These results indicate that the genetic manipulations in this study did not affect the growth of parasite *in vitro*.

The proliferation of *Babesia* organisms in the vectors is an essential part of their survival. However, the detailed life-cycle of the parasite in ticks, including information about the timing of migration, remains unknown [20]. *Haemaphysalis longicornis*, a vector for *B. gibsoni* [21], is widely used as a model tick to study pathophysiology in tick infestation [22]. Therefore, transfected *B. gibsoni* and *H. longicornis* could be used for developing tick-*Babesia* experimental models for clarifying the kinetics of the tick stage of canine *Babesia* parasites. A tick-*Babesia* interactions model may contribute to a better understanding of tick transmission as well as the way *Babesia* species interact with the ticks.

All previously established transfection systems for *Babesia* focused on bovine *Babesia* species, which were transfected using Gene Pulser Xcell™ Electroporation system (Bio-Rad, VA, USA) and AMAXA Nucleofector™ 2b device (Lonza) [10–12]. However, these transfection systems were not effective for *B. gibsoni* [13]. Therefore, the present method based on 4D Nucleofector™ may provide a more suitable transfection system for non-bovine *Babesia* parasites, such as *B. gibsoni*. The rapid *in vitro* aging of canine erythrocytes [23] may play an important role in restricting a successful transfection. Therefore, to avoid the rapid aging of canine erythrocytes, we strongly suggest subculturing every week by fresh RBCs after transfection. A host-*Babesia* infection model may be easier to achieve using canine *Babesia* rather than bovine *Babesia* because using dogs for animal experiments is more feasible than using cattle. The urgently needed genome edited host-*Babesia* infection model may help us monitor transmission *in vivo*, investigate mechanisms of infection and immunity, and also improve the development of novel strategies for controlling babesiosis.

## Conclusions

In summary, we established a stable transfection system for *B. gibsoni* and successfully integrated exogenous genes into the *B. gibsoni* genome. The establishment of this system is critical to fulfill genome editing, which may contribute to determining gene function, discovery of novel drug targets, establishment of infection model and evaluation of the interactions between the parasite and the host.

## Additional files

Additional file 1: **Figure S1.** *Babesia gibsoni* sensitivity to WR99210. All data are expressed as means ± SD of triplicate cultures. (PPTX 100 kb)

Additional file 2: **Figure S2.** Growth curves of wild type (WT) and genome integrated (GI) parasites. WT and GI parasites were maintained by sub-culturing every 3 days and parasitemia were monitored daily. All data are expressed as means ± SD of triplicate cultures. (PPTX 69 kb)

## Abbreviations

*ef-1α*: *Elongation factor-1 alpha*
GFP: Green fluorescent protein
GI: Genome integrated
*hdhfr*: *Human dihydrofolate reductase*
IG: Intergenic region
iRBCs: Infected red blood cell
ORF: Open reading frame
PCR: Polymerase chain reaction
*rap-1*: *Rhoptry associated protein-1*
RBCs: Red blood cell
RT: Room temperature
SD: Standard deviation
WT: Wild type
