# Supplementary figures and images for: Establishment of a stable transfection system for genetic manipulation of Babesia gibsoni
Source: Parasit Vectors. 2018 Apr 23;11:260. doi: 10.1186/s13071-018-2853-1 (PMC5914073; doi:10.1186/s13071-018-2853-1)

## Slide 1
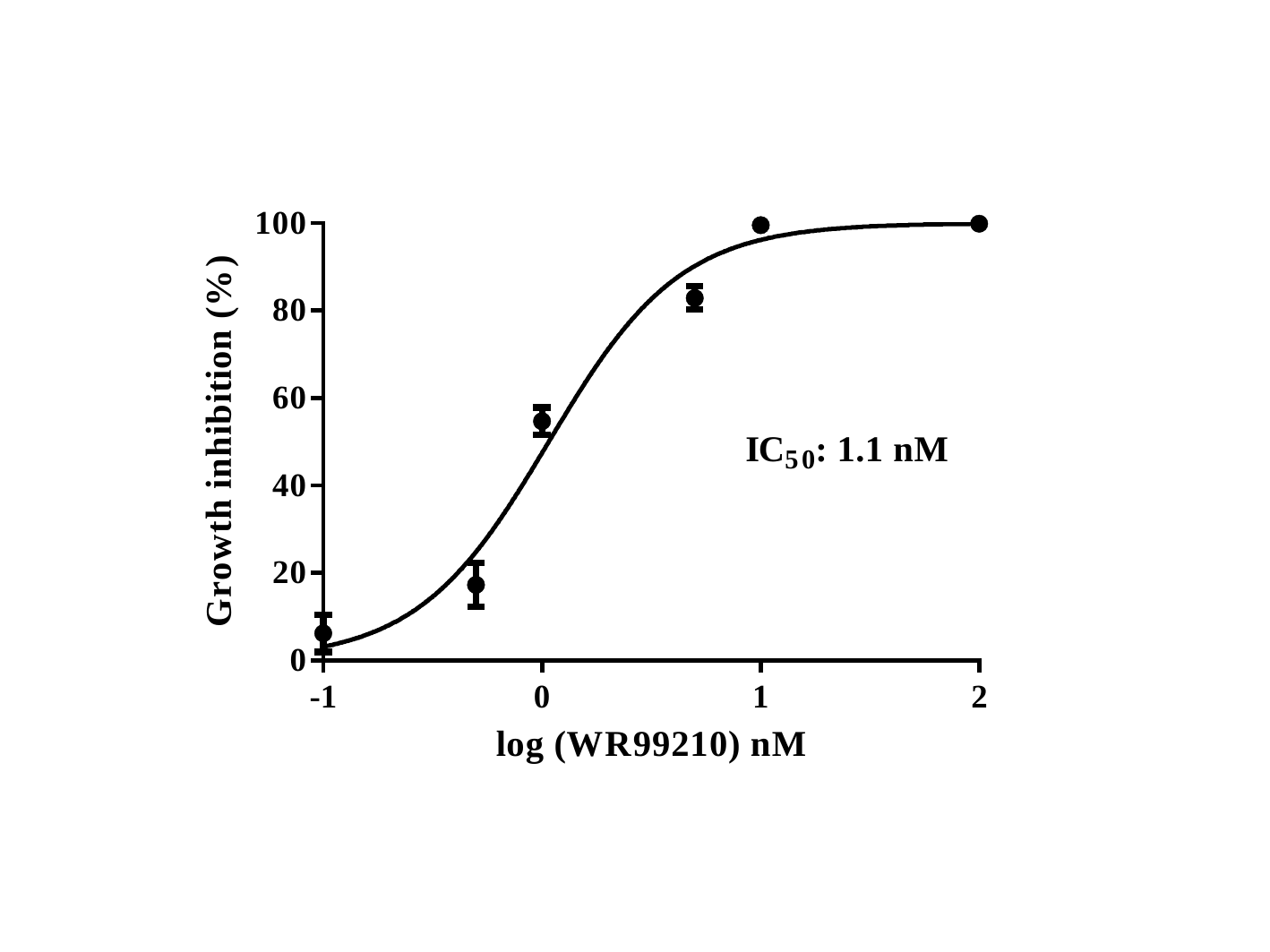

Supplement: Supplementary file 1 — Figure S1. Babesia gibsoni sensitivity to WR99210. All data are expressed as means ± SD of triplicate cultures. (PPTX 100 kb) [file 13071_2018_2853_MOESM1_ESM.pptx]

## Slide 1
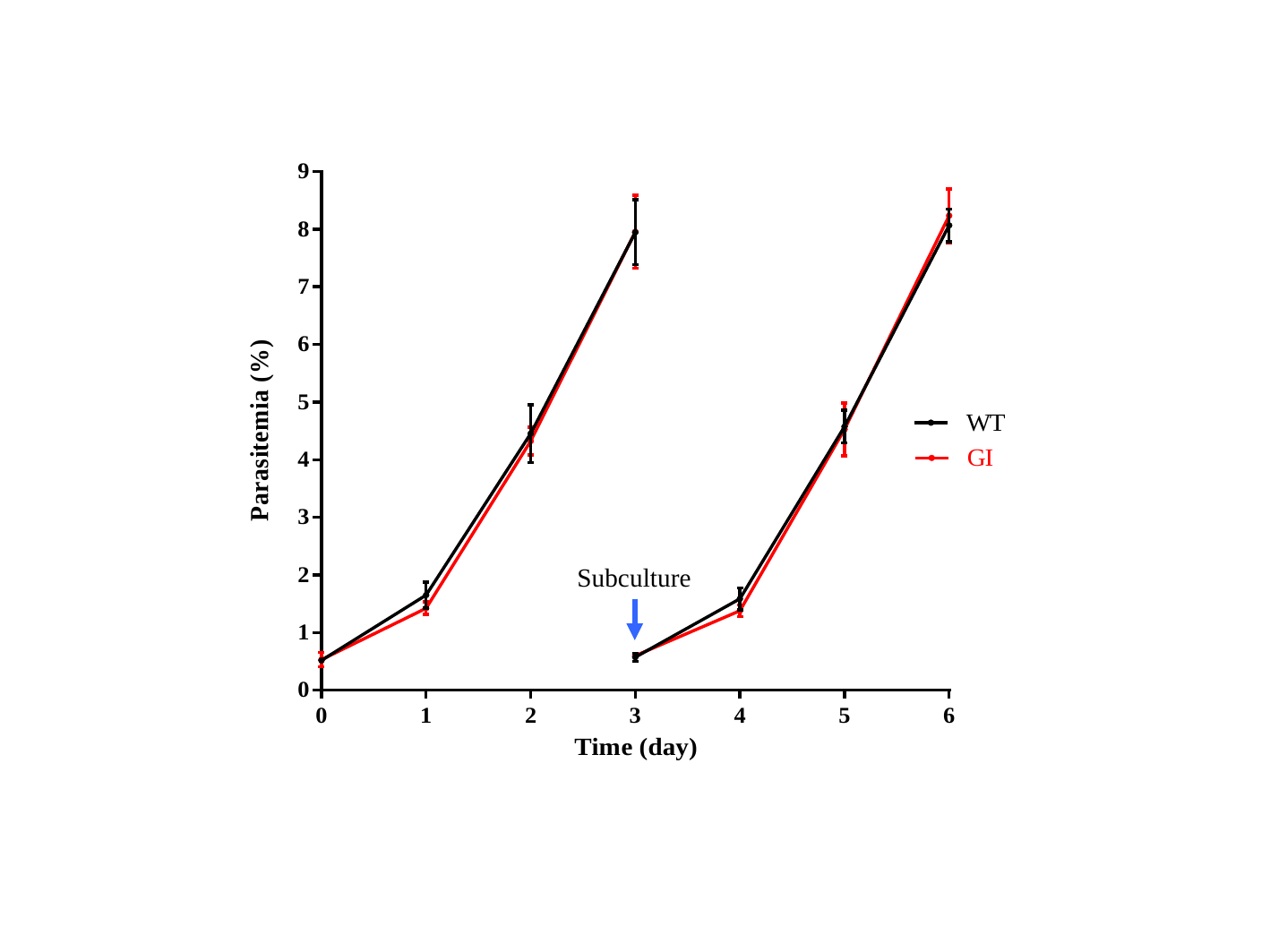

Subculture

Supplement: Supplementary file 2 — Figure S2. Growth curves of wild type (WT) and genome integrated (GI) parasites. WT and GI parasites were maintained by sub-culturing every 3 days and parasitemia were monitored daily. All data are expressed as means ± SD of triplicate cultures. (PPTX 69 kb) [file 13071_2018_2853_MOESM2_ESM.pptx]
